# Modular and mechanistic changes across stages of colorectal cancer

**DOI:** 10.1186/s12885-022-09479-3

**Published:** 2022-04-21

**Authors:** Sara Rahiminejad, Mano R. Maurya, Kavitha Mukund, Shankar Subramaniam

**Affiliations:** 1 Department of Bioengineering, University of California, San Diego, La Jolla, CA USA; 2 Department of Mechanical and Aerospace Engineering, University of California, San Diego, La Jolla, CA USA; 3 San Diego Supercomputer Center, University of California, San Diego, La Jolla, CA USA; 4 Department of Cellular and Molecular Medicine, University of California, San Diego, La Jolla, CA USA; 5 Department of Computer Science and Engineering, University of California, San Diego, La Jolla, CA USA

**Keywords:** Colorectal cancer, CRC stages, Stage-specific networks, Stage-unique networks, Biomarkers, Signaling pathways

## Abstract

**Background:**

While mechanisms contributing to the progression and metastasis of colorectal cancer (CRC) are well studied, cancer stage-specific mechanisms have been less comprehensively explored. This is the focus of this manuscript.

**Methods:**

Using previously published data for CRC (Gene Expression Omnibus ID GSE21510), we identified differentially expressed genes (DEGs) across four stages of the disease. We then generated unweighted and weighted correlation networks for each of the stages. Communities within these networks were detected using the *Louvain* algorithm and topologically and functionally compared across stages using the normalized mutual information (NMI) metric and pathway enrichment analysis, respectively. We also used Short Time-series Expression Miner (STEM) algorithm to detect potential biomarkers having a role in CRC.

**Results:**

Sixteen Thousand Sixty Two DEGs were identified between various stages (*p*-value ≤ 0.05). Comparing communities of different stages revealed that neighboring stages were more similar to each other than non-neighboring stages, at both topological and functional levels. A functional analysis of 24 cancer-related pathways indicated that several signaling pathways were enriched across all stages. However, the stage-unique networks were distinctly enriched only for a subset of these 24 pathways (e.g., MAPK signaling pathway in stages I-III and Notch signaling pathway in stages III and IV). We identified potential biomarkers, including *HOXB8* and *WNT2* with increasing, and *MTUS1* and *SFRP2* with decreasing trends from stages I to IV. Extracting subnetworks of 10 cancer-relevant genes and their interacting first neighbors (162 genes in total) revealed that the connectivity patterns for these genes were different across stages. For example, *BRAF* and *CDK4*, members of the Ser/Thr kinase, up-regulated in cancer, displayed changing connectivity patterns from stages I to IV.

**Conclusions:**

Here, we report molecular and modular networks for various stages of CRC, providing a pseudo-temporal view of the mechanistic changes associated with the disease. Our analysis highlighted similarities at both functional and topological levels, across stages. We further identified stage-specific mechanisms and biomarkers potentially contributing to the progression of CRC.

**Supplementary Information:**

The online version contains supplementary material available at 10.1186/s12885-022-09479-3.

## Background

Colorectal cancer (CRC) refers to cancers affecting both colon and rectum. According to GLOBOCAN 2020 data, CRCs are the third most diagnosed and the second most deadly form of cancer worldwide, comprising 11% of all cancer diagnoses [1]. The survival is highly dependent upon the stage of disease at diagnosis and earlier detection portends higher chance of survival [2]. Two types of risk factors contribute to the incidence of CRC. The first type includes the ones that are beyond the control of the individual, such as age and hereditary factors. The second type is related to environmental and lifestyle risk factors such as diets high in fat, physical inactivity, smoking, and heavy alcohol consumption [3].

CRC is said to progress through five stages. The earliest stage, stage 0 represents the presence of abnormal cells in the mucosa of the colon wall. In stage I, tumor penetrates the submucosa of the colon or rectum wall, while at stage II the cancer has spread through the wall to the serosa, but not the nearby organs. Stage III represents cancer in the mucosa, submucosa, serosa and the spread into the nearby lymph nodes. Stage IV represents the most aggressive form of CRC, where the cancer metastasizes and spreads to other parts of the body [4]. Biomarkers, agnostic of stages, have been used for detection of CRC [5]. For example, *p53*, a key biomarker, is a tumor suppressor gene, mutated in 34% of the proximal colon tumors and in 45% of the distal colorectal tumors [6, 7]. Prior work from our group [8] and many others have identified potential causes and mechanisms of CRC, but a few have focused on identifying the stage-specific dysregulation, and biomarkers. Palaniappan et al. identified novel cancer genes that could underlie the stage-specific progression and metastasis of CRC [9]. Cai et al. performed a comprehensive untargeted metabolomics analysis on normal and tumor colon tissues from CRC patients and identified 28 highly discriminatory tumor tissue metabolite biomarkers [10].

**Fig. 1:**
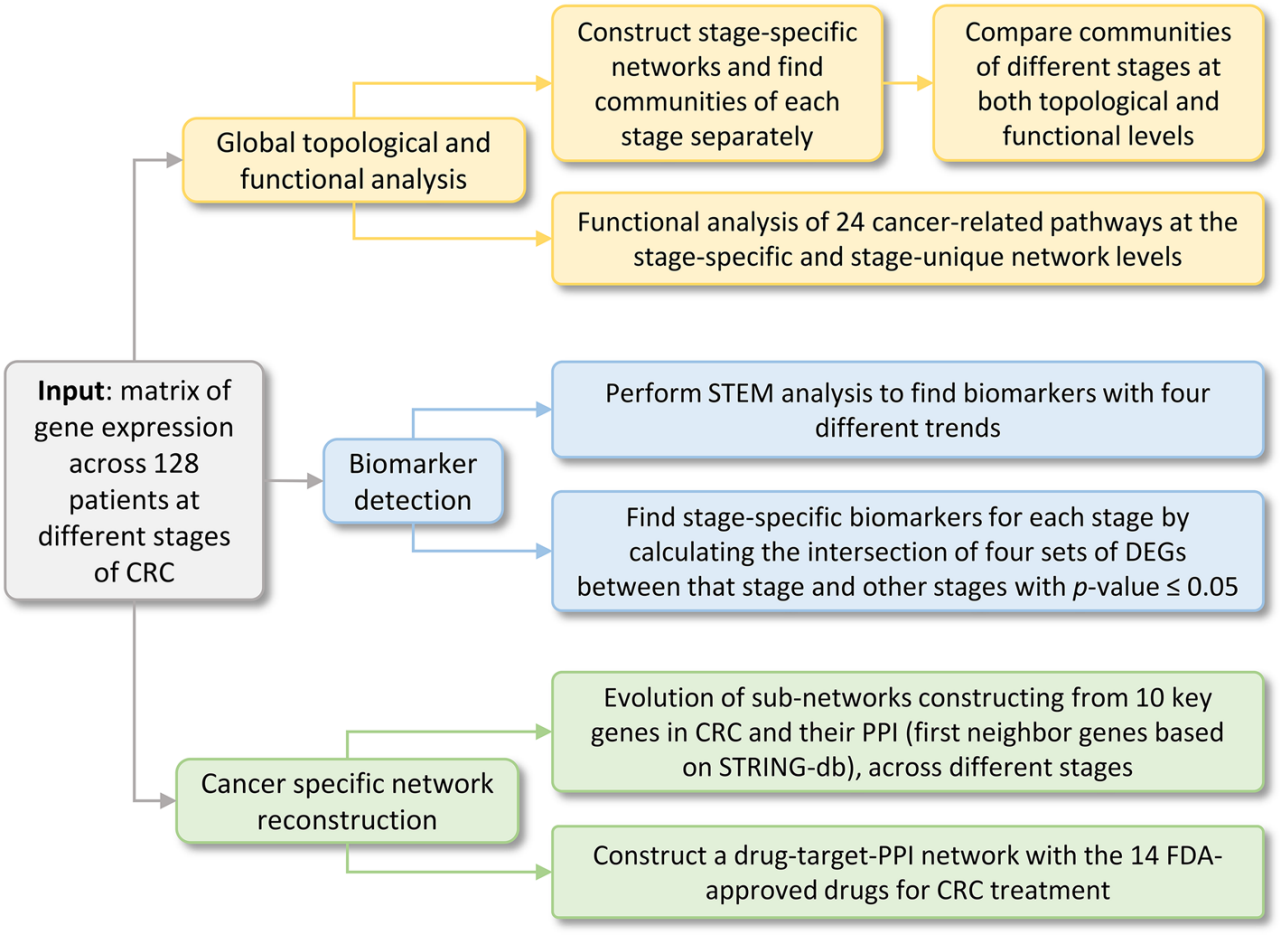
Flow chart of the approach used in our analysis

In this study, we focused on modelling each stage as a molecular network and identifying subnetworks (communities) which enable better mechanistic interpretation [11, 12]. To this extent, we utilized a gene expression microarray dataset containing 104 human CRC samples (across stages I to IV) and 24 normal samples from Gene Expression Omnibus (GEO) to detect stage-specific biomarkers and modular mechanisms, potentially causal for the progression of CRC. We first constructed gene correlation networks for each of the stages, and detected communities using the *Louvain* algorithm [13, 14]. The communities are functionally interpreted in the context of CRC. We also developed stage-unique networks (by retaining edges unique to that specific stage) and functionally interpret them. Next, we utilized Short Time-series Expression Miner (STEM) approach to identify candidate biomarkers with substantial/monotonic changes across stages [15, 16]. A biologically driven analysis enabled characterization of the evolution of molecular subnetworks across stages. Lastly, a drug-target-PPI (Protein–Protein Interaction) network is generated which may provide insight into understanding stage-specific functional mechanisms for some of the current drugs used in CRC treatment. Figure 1 shows a flow chart for our analysis pipeline.

## Materials and methods

### Microarray data pre-processing

We used a CRC microarray dataset from the GEO (accession ID GSE21510) containing samples from 13 patients in stage I, 37 patients in stage II, 34 patients in stage III, and 20 patients in stage IV cancer along with 24 normal samples. There was only one sample associated with stage 0 and we excluded it from our analysis. More details about the clinical characteristics of the GSE21510 have been presented in the original publication by Tsukamoto et al. [17]. The raw dataset had 54,675 probe IDs across 128 samples/patients and it was re-normalized using Robust Multi-array Average (RMA) normalization [18]. Probe IDs with missing or multiple Entrez gene IDs (based on annotation file from GEO) were removed from the dataset. Both linear and non-linear dimensionality reduction algorithms (Principal Component Analysis (PCA) [19] and t-Distributed Stochastic Embedding (t-SNE) [20]) were used to detect outliers in the data. PCA was performed in R, using *prcomp* and *autoplot* functions (of *ggfortify* package). t-SNE was also performed in R, using *Rtsne* package.

### Differentially expressed genes (DEGs)

We identified DEGs at the probe level using *limma* [21] between each pair of neighboring stages (i.e., stage I vs. normal, stage II vs. I, stage III vs. II and stage IV vs. III). For genes with multiple probe IDs, the geometric mean of the *p*-values of the multiple probes was used as the *p*-value for the gene. DEGs were then identified at *p*-value ≤ 0.05 for each comparison and their union across 4 comparisons (stage I vs. normal, stage II vs. I, stage III vs. II and stage IV vs. III) was calculated as a master list of DEGs.

### Network construction

Networks for each of the stages were constructed using correlation of gene expression values for the DEGs identified. Specifically, the Pearson Correlation Coefficient (PCC) [-1, 1], *r*, was calculated between all gene-pairs. Networks were then constructed using a cut-off for PCC at each stage (*r*_th_) based on the degree of freedom (number of patients at that specific stage - 2) and a *p*-value threshold of 0.001. Edges with *p*-value ≤ 0.001 (i.e., |r|≥ *r*_th_) were retained. The weight of edges was binary (0 or 1) for unweighted networks and non-binary (0 ≤ *w* ≤ 1) for weighted networks, with the absolute value of PCC being used as weights. We refer to these networks as stage-specific networks (whole networks for the normal, stage I, II, III, and IV). Stage-unique networks were also constructed for each of the stages by removing edges from stage-specific network of each stage that were common with any other stage-specific network.

### Community detection

We used the *Louvain* algorithm to detect communities within each stage-specific network given its established status as the leading method for community detection [13, 14]. *Louvain* detects network communities by maximizing modularity (a measure of the density of links (edges) within communities compared to links between communities). Briefly, the search for communities using the algorithm proceeds in two phases. During the first phase, communities are detected by optimizing modularity locally. During the second phase, nodes of the same community are aggregated as pseudo-nodes to generate a new network. The combination of these two phases is iterated, until the modularity reaches a local maximum. The computational complexity of this algorithm is (*O*(*nlogn*)) which makes it extremely fast [14] (also see Supplementary Methods).

### Topological and functional comparison of communities

Normalized Mutual Information (NMI) metric was utilized to compare communities of different stages at a topological level [22]. NMI is 1 when a network is compared with itself. Larger (smaller) the value of NMI, more (less) similar are the networks being compared (see Supplementary Methods). To assess the statistical significance of the NMI values, we needed to compute their *p*-value. Hence, we generated 1000 random networks with the same number of nodes, edges and degree distribution as the stage I-, II-, and III-specific networks. Communities of random networks were identified using the *Louvain* algorithm and compared between stage I- and II- and stage II- and III-specific networks using the NMI metric. *p*-values for comparing the stage-specific networks were then calculated from the histogram of the 1000 NMI values.

Jaccard index (JI), the ratio of the count of common genes to the count of union of genes in two groups, was used to identify pairs of communities which were similar to each other in terms of genes common between them. The most similar communities were then compared at a functional level. We used Kyoto Encyclopedia of Genes and Genomes (KEGG) pathway enrichment available via DAVID version 6.8 [23, 24] for functional analysis [25].

### Edge-based functional enrichment

*p*-values for edge-based enrichment were computed using a hypergeometric test for edges (gene pairs) [26] which accounted for the topology of the network. For *d* DEGs, the total number of edges, *N*, is calculated as *d*(*d* - 1)/2. Similarly, for a given KEGG pathway with *d*_*KEGG*_ enriched genes (from our master list of *d* DEGs), *m*_*KEGG*_ edges are calculated (*m*_*KEGG*_ = *d*_*KEGG*_(*d*_*KEGG*_ - 1)/2). Suppose a network contains *n* edges, of which *k* edges are between *d*_*KEGG*_ genes of the given KEGG pathway, then a *p*-value for the edge-based enrichment of this pathway is calculated from a hypergeometric distribution as:

$$p\left(k|KEGG pathway\right)=\sum_{i=k}^{{m}_{KEGG}}P(X=i|KEGG pathway)$$

1 $$=\sum_{i=k}^{{m}_{KEGG}}\frac{\left(\genfrac{}{}{0pt}{}{{m}_{KEGG}}{i}\right)\left(\genfrac{}{}{0pt}{}{N-{m}_{KEGG}}{n-i}\right)}{\left(\genfrac{}{}{0pt}{}{N}{n}\right)}$$

Equation 1 provides an estimate for the probability of observing *k* or more edges between *p*_*KEGG*_ genes for the given KEGG pathway [26]. The R function *phyper* with 4 parameters was used to calculate the edge-based *p*-value using *phyper* (*k* - 1, *n*, *N* - *n*, *m*_*KEGG*_, *lower.tail* = *FALSE*).

### Biomarker identification

The STEM algorithm [15, 16] (see Supplementary Methods) was utilized to identify potential biomarkers. Since there were different number of patients at each stage, the median of gene-expression for patients at each stage was considered as the representative gene expression for that stage. STEM works by first selecting a set of potential profiles and then assigning genes to the profile that best captures their expression trend. We selected 60 model profiles and a maximum unit change of 1, which represents the change a gene could have between successive time points. Gene Expression Profiling Interactive Analysis (GEPIA2) [27] was next used to validate the biomarkers identified using STEM analysis within independent cohort (TCGA COAD-READ) at |log2FC|≥ 1 and q-value (FDR adjusted *p*-value) ≤ 0.05.

### Supervised analysis with key genes

We identified the interacting proteins of 10 key genes with known roles in CRC using STRING-db [28]. A subnetwork of the key genes and their first neighbors were extracted from each stage-specific network, separately. The analysis consisted of the following steps:

- Detection of the first neighbors of 10 key genes from STRING-db with two criteria: score threshold ≥ 0.4 and up to 20 connections between genes.
- Identification of unique genes from the union of 10 key genes and their first neighbors found in STRING-db.
- Extraction of subnetworks of the unique genes from each stage-specific network and their visualization using Cytoscape [29]. |PCC| between the genes were used as edge-weights.

### Drug-target-PPI network

Approved drugs and their target genes for CRC were identified from National Cancer Institute (NCI) [30] and DrugBank databases [31]. We then projected the PPI information from STRING-db (score threshold ≥ 0.9) [28] and gene weights from the stage-specific networks onto the drug-target interactions detected above. We also identified important KEGG pathways related to these target genes. The constructed network was visualized using Cytoscape [29].

## Results and discussion

### Identification of DEGs

The CRC dataset used here contained 41,834 probe IDs across 128 samples after pre-processing (see Materials and Methods). Outlier detection using PCA and t-SNE identified two normal samples as outliers which were eliminated, leaving 126 samples for our analysis. The first two PCs and the first two dimensions of t-SNE are shown in Additional file 2: Figures S1A and S1B, respectively. In order to capture the most significant genes, we identified DEGs (see Materials and Methods) between neighboring stages with *p*-value ≤ 0.05 resulting in 15,634 DEGs between stage I and normal, 528 DEGs between stages II and I, 745 DEGs between stages III and II, and 503 DEGs between stages IV and III. The union of all DEGs (16,062 unique genes) was considered as the master list of DEGs for all downstream analysis.

### Correlation-based network analysis

PCC was calculated for all pairs of DEGs to construct the networks (see Materials and Methods). For each stage, based on the number of patients and a fixed *p*-value, we identified the corresponding threshold for PCC. Unweighted, stage-specific and stage-unique networks were subsequently constructed using the 16,062 DEGs [32]. Table 1 lists some basic properties for different stage-specific networks. Properties for unweighted networks are listed in Additional file 1: Table S1. Number of nodes and edges for all communities of stage-specific and unweighted networks can be found in Additional file 1: Tables S2 and S3, respectively.

**Table 1:** Properties for stage-specific networks

Network	# of patients	PCC cut-off	# of edges	# of communities	Modularity
Normal	22	0.6523	1,809,792	18	0.43
Stage I	13	0.8009	507,603	17	0.51
Stage II	37	0.5186	1,063,390	9	0.44
Stage III	34	0.5392	1,214,109	9	0.45
Stage IV	20	0.6788	763,554	11	0.45

Stage-specific networks of different stages with the number of patients listed in the second column, PCC cut-off for *p*-value 0.001 in the third column, number of edges in the fourth column, number of communities and modularity scores in the last two columns

In the following section, we compare communities detected within stage-specific networks at the topological and functional levels. The NMI metric was used to compare networks at a topological level. Highly similar networks (at the topological level) were further analyzed at a functional level. KEGG pathway enrichment analysis was used to assess functional similarity of the communities detected.

#### Neighboring stages are functionally and topologically similar

**Fig. 2:**
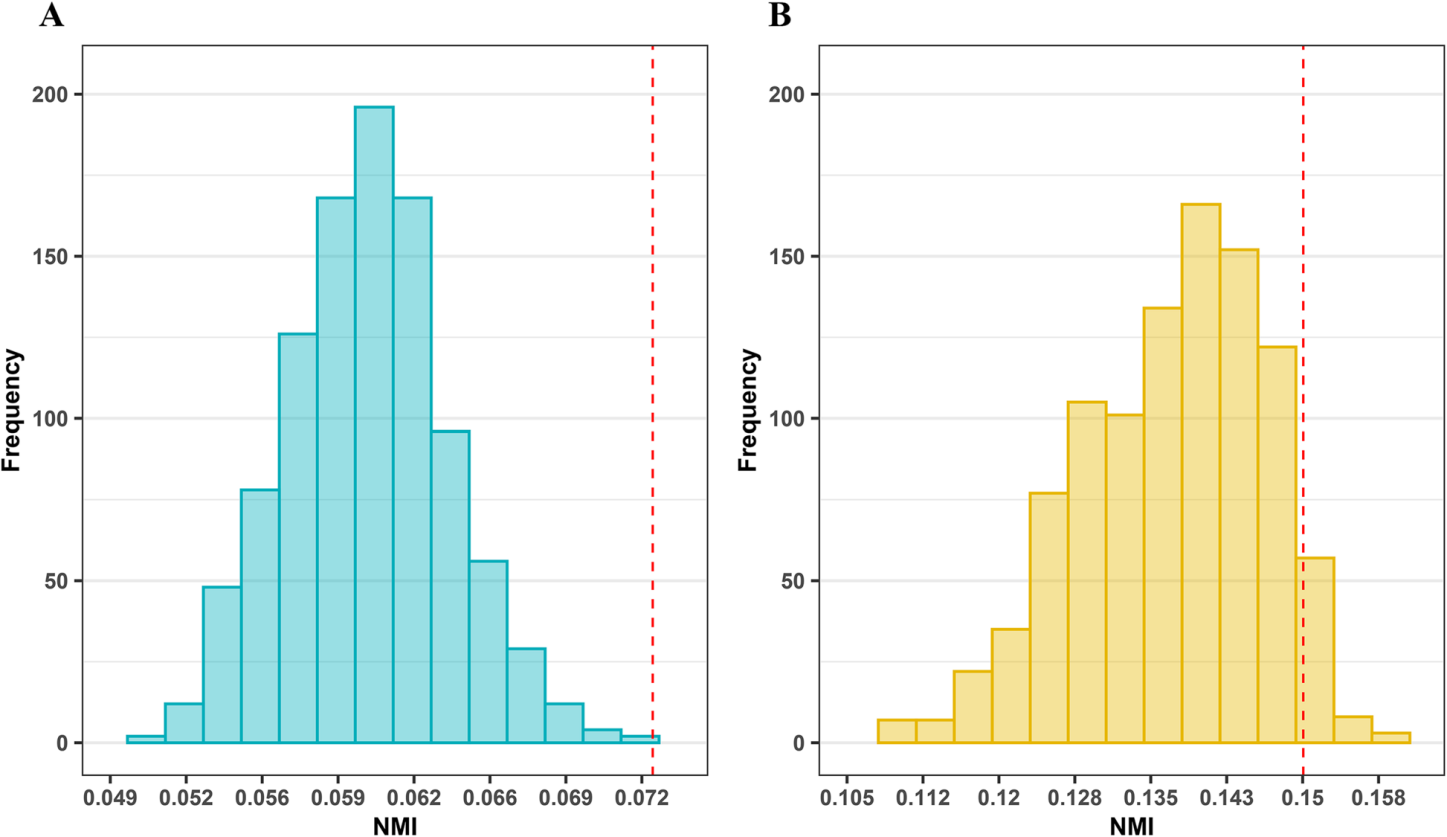
Histogram of a permutation test for comparing communities of different stages with degree preservation. **A** NMI metric between random networks with sizes equal to stage I- and II-specific networks. The actual value of NMI for comparing those stages is 0.0729 (vertical dotted line), corresponding to a *p*-value of 0.001 (significant for a *p*-value threshold of 0.05). **B** NMI metric between random networks with sizes equal to stage II- and III-specific networks. The actual value of NMI for comparing those stages is 0.1501 (vertical dotted line), (*p*-value of 0.05, significant)

Using the NMI metric we evaluated the similarity between networks. Table 2 and Additional file 1: Table S4 represent the results of comparing communities of stage-specific networks and unweighted networks using NMI. Based on the results of Table 2 (and Additional file 1: Table S4), neighboring stages were found to be more similar to each other than non-neighboring stages. A permutation test was also performed to assess the statistical significance of the NMI values seen in Table 2 (see Materials and Methods). Figures 2A and 2B show histograms for the values of NMI between communities of the random networks of stages I and II, and II and III, respectively. Our analysis highlighted that the NMI calculated between stage-specific networks was highly significant (e.g., *p*-value of NMI between communities of the stages I- and II-specific networks was 0.001 and between communities of the stages II- and III-specific networks was 0.05).

**Table 2:** Comparing stage-specific networks using NMI

	Normal	Stage I	Stage II	Stage III	Stage IV
Normal	1	0.0282	0.0403	0.0444	0.0276
Stage I		1	0.0729	0.0689	0.045
Stage II			1	0.1501	0.0887
Stage III				1	0.0771
Stage IV					1

NMI is 1 (diagonal elements) when a network is compared with itself. Larger (smaller) the value of NMI, the more (less) similar are the two networks being compared. For example, the network of stage I is more similar to the network of stage II than to the networks of stages III or IV

**Fig. 3:**
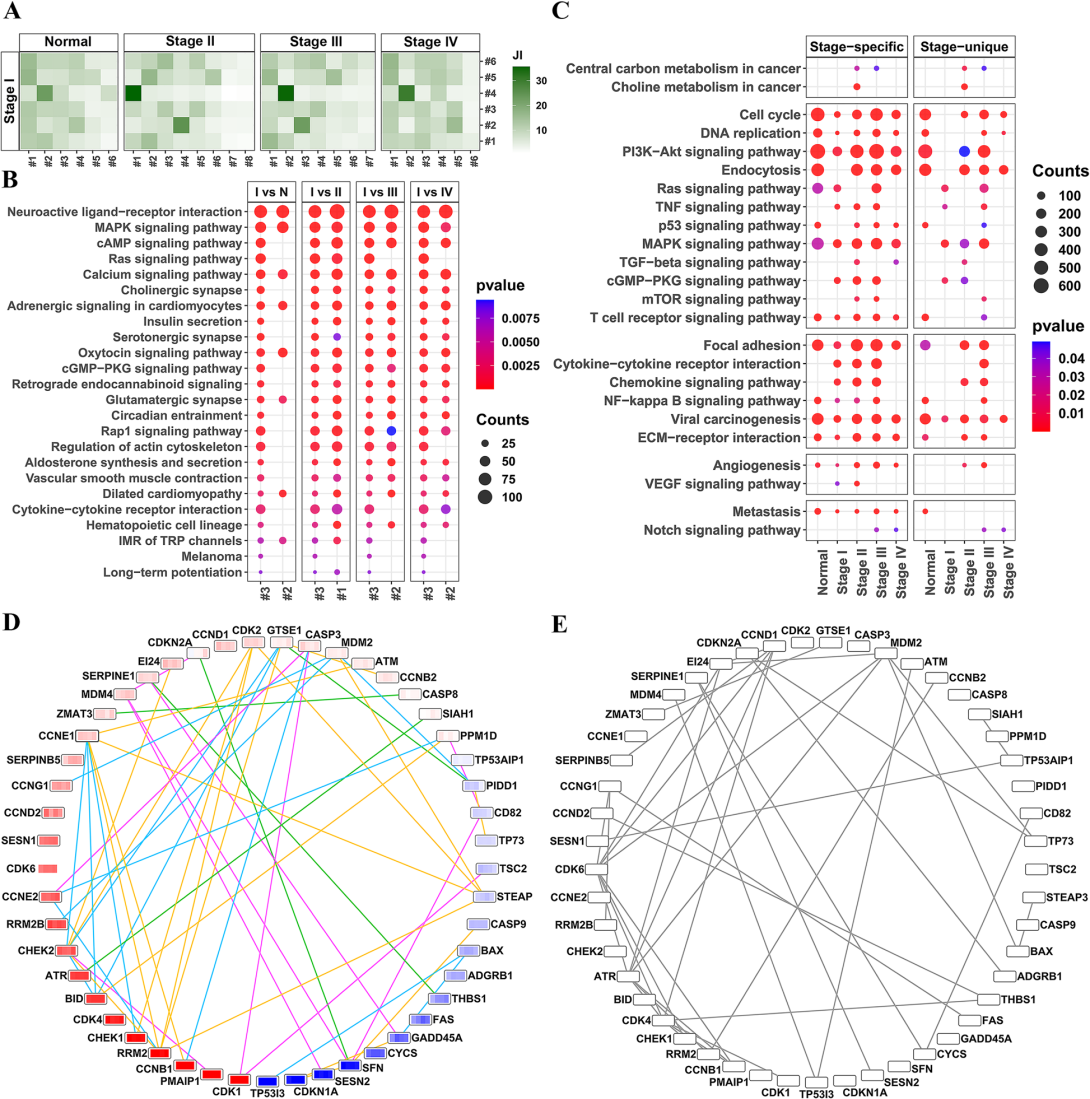
Topological and functional analysis of the weighted correlation networks. **A** Heat map for JI values for comparing the communities of stage I-specific network with the communities of other stages. The color-scale is from white for the minimum value of JI (0%) to green for the maximum value (36%). The largest value in each table is selected to perform the functional enrichment. **B** Functional comparison of the KEGG pathways with *p*-values ≤ 0.01 and having more than 10 genes for the third community of stage I-specific network with the corresponding communities of other stages. The third community of stage I is very similar to the first community of stage II in terms of the gene counts and *p*-values of the enriched pathways but less similar to the second community of normal, stages III and IV. **C** Functional comparison (edge-based enrichment) of the stage-specific and stage-unique networks for 24 cancer-related pathways. The pathways are divided into five categories: cancer related, cell cycle/proliferation/growth, inflammation, angiogenesis and metastasis. Functional enrichment is carried out for both “stage-specific” and “stage-unique” networks. Number of edges related to the genes enriched in each pathway are indicated by the size of the dots. Color scale of the dots indicate *p*-value with a cut-off of 0.05. **D** Connectivity of p53 signaling pathway genes across different stage-unique networks. The nodes are colored based on the log2FC values (in a specific stage vs. normal) across the four stages I-IV (dark blue (log2FC of -2) to white (0) to dark red (2)). Each node represents four log2FC values, going from left to right. Edges are colored differently across stages as follows: green for edges in stage I, cyan in stage II, yellow in stage III, and purple in stage IV. **E** Connectivity of p53 signaling pathway genes in normal

We next used JI values for direct topological comparison of individual communities across stage-specific networks. The higher the value of JI, the more similar were the communities being compared. For example, JI values for comparing the communities of stage I-specific network with the communities of other stages is shown in Fig. 3A (see also Additional file 1: Table S5). Likewise, a functional comparison of the third community of stage I with the corresponding communities of other stages revealed that the third community of stage I was more similar to the first community of stage II (the neighboring stage) than the corresponding communities of other stages (based on JI) (see Fig. 3B and Additional file 1: Tables S6 through S9). Pathways indicated in this comparison were chosen at a *p*-value ≤ 0.01 and with more than 10 genes from the third community of stage I. For some pathways such as Ras signaling pathway, the number of enriched genes and the *p*-values were similar between the third community of stage I and the first community of stage II but did not meet the threshold for the corresponding communities of other stages.

Analyzing each community individually can leave out important functions due to the distribution of functionally related genes between them. Hence, we carried out an edge-based functional analysis (see Materials and Methods) at the whole network level (consisting of 16,062 genes). We constructed stage-unique networks and compared both types of networks (stage-specific and stage-unique) at a functional level.

#### Functional analysis at the whole network level

Some of the edges were common among two or more stage-specific networks. To identify edges unique to each stage, we constructed stage-unique networks (See Materials and Methods). We identified 1,668,692 edges for normal-, 430,446 edges for stage I-, 839,058 edges for stage II-, 967,358 edges for stage III-, and 627,558 edges for stage IV-unique networks. The number of edges for the stage-specific networks are listed in Table 1.

To ascertain the functional relevance for the networks, we first selected 24 pathways associated with cancer progression (from initiation to metastasis) and carried out a supervised analysis. A list of all pathways enriched for the master list of genes can be found in Additional file 1: Table S10. We calculated the edge-based *p*-values and performed an enrichment for the 24 pathways for the stage-specific (see Table 1) and stage-unique networks (see Fig. 3C). The *p*-value cut-off was 0.05. The number of edges associated with genes enriched in the stage-unique networks were less than the stage-specific networks for all stages and for all 24 pathways. We noted that the number of edges for each stage-unique network was less than its value for the stage-specific network of that stage. For example, stage I-unique network had 430,446 edges as compared to the stage I-specific network with 507,603 edges.

Among the 24 cancer-related pathways, we observed that central carbon metabolism pathway was enriched across stages II and III and is known to play a role in cancer progression [33]. Cell cycle and DNA replication pathways were significantly enriched in almost all stages with more edges in the Cell cycle pathway. Several signaling pathways including PI3K-Akt, Ras, MAPK, TGF-beta, p53, and T cell receptor signaling pathway associated with cell growth were enriched across stages. PI3K-Akt signaling pathway plays an important role in the growth and progression of CRC. Both MAPK and PI3K-Akt serve as a molecular target for treatment of CRC [34, 35]. TGF-beta signaling pathway was particularly enriched only in stages II, and IV. TGF-beta is known to play a significant role in inflammation and tumorigenesis by modulating cell growth, differentiation, apoptosis, and homeostasis, contributing to tumor maintenance and cancer progression [36]. Besides changes in enrichment of specific pathways, changes in connectivity pattern of specific genes in key pathways were also observed across the CRC stages. For example, Figs. 3D and 3E show the connectivity pattern of genes in the p53 signaling pathway across stages I-IV and normal, respectively. p53 signaling pathway has a critical role in the regulation of Cell cycle, DNA replication and apoptosis [37]. Comparing Figs. 3D and 3E revealed that hub genes were different between cancer stages and normal. For example, *CCND1* and *CDK6* were two genes with high connectivity (degree) in normal only. *CCND1* is a proto-oncogene which is known to play a critical role in promoting the G1-to-S transition of the cell cycle in many cell types [38]. Likewise, *CCNE1*, also a proto-oncogene, displayed high degree of connectivity in stages II and III which was not present in normal. *CCNE1* serves as a positive regulator of cell cycle and promotes G1-to-S phase transition by activating *CDK2* [39, 40]. *CDK2* also showed high degree of connectivity in stage III, although it was not present in normal.

Focal adhesion pathway was more enriched in normal, stage II and stage III than in other stages. Focal adhesion kinase (*FAK* or *PTK2*) is a major integrin-dependent tyrosine phosphorylated protein in this pathway and known to contribute significantly to inflammatory signaling pathways. *PTK2* has been suggested to be a potential target for CRC therapies [41]. NF-kappa B signaling pathway was enriched in normal, and stages I, II and III, and is a regulator of immune response and inflammation and associated with carcinogenesis [42]. VEGF signaling associated genes, with known roles in angiogenesis and metastasis, were enriched in stages I and II [43], while Notch signaling pathway, a main pathway in metastasis and tumor angiogenesis processes, was enriched in stages III and IV.

Overall, most of the cancer related pathways were enriched across all stage-specific networks. However, the enrichment of those pathways was distinct across stage-unique networks.

#### *In-silico* validation

To validate our result at the gene and pathway level, we analyzed the TCGA COAD-READ data available through GEPIA2 by identifying DEGs with q-value < 0.05 for COAD and READ cohorts, resulting in 16,438 DEGs common to both. Since GEPIA2 does not allow for stage-wise identification of DEGs, we calculated DEGs across all stages (104 samples) and normal (22 samples) at q-value < 0.05 within our dataset. A total of 16,641 DEGs were identified, of which 11,389 were common with the TCGA COAD-READ cohort. A hypergeometric test on the overlap indicated that the number of DEGs as common were statistically significant (*p* = 0.05). The total number of genes used for the hypergeometric test was 24,136. The log2FC of genes identified as common between the COAD, READ and our dataset are also provided in Additional file 1: Table S11. Of the 11,389 genes, ~ 65% of the genes showed expression trends in the same direction within COAD-READ as DEGs identified in our current study. Functional analysis of the 11,389 genes further revealed several signalling pathways enriched crucial to CRC consistent with our results including Ras, MAPK, PI3K-AKT, TGF-beta and WNT signalling (Fig. 3C).

### Biomarkers

We performed STEM analysis to identify potential biomarkers and validated them using TCGA COAD-READ cohort, available through GEPIA2 [27].

#### Four distinct biomarker trends identified in CRC via STEM analysis

**Fig. 4:**
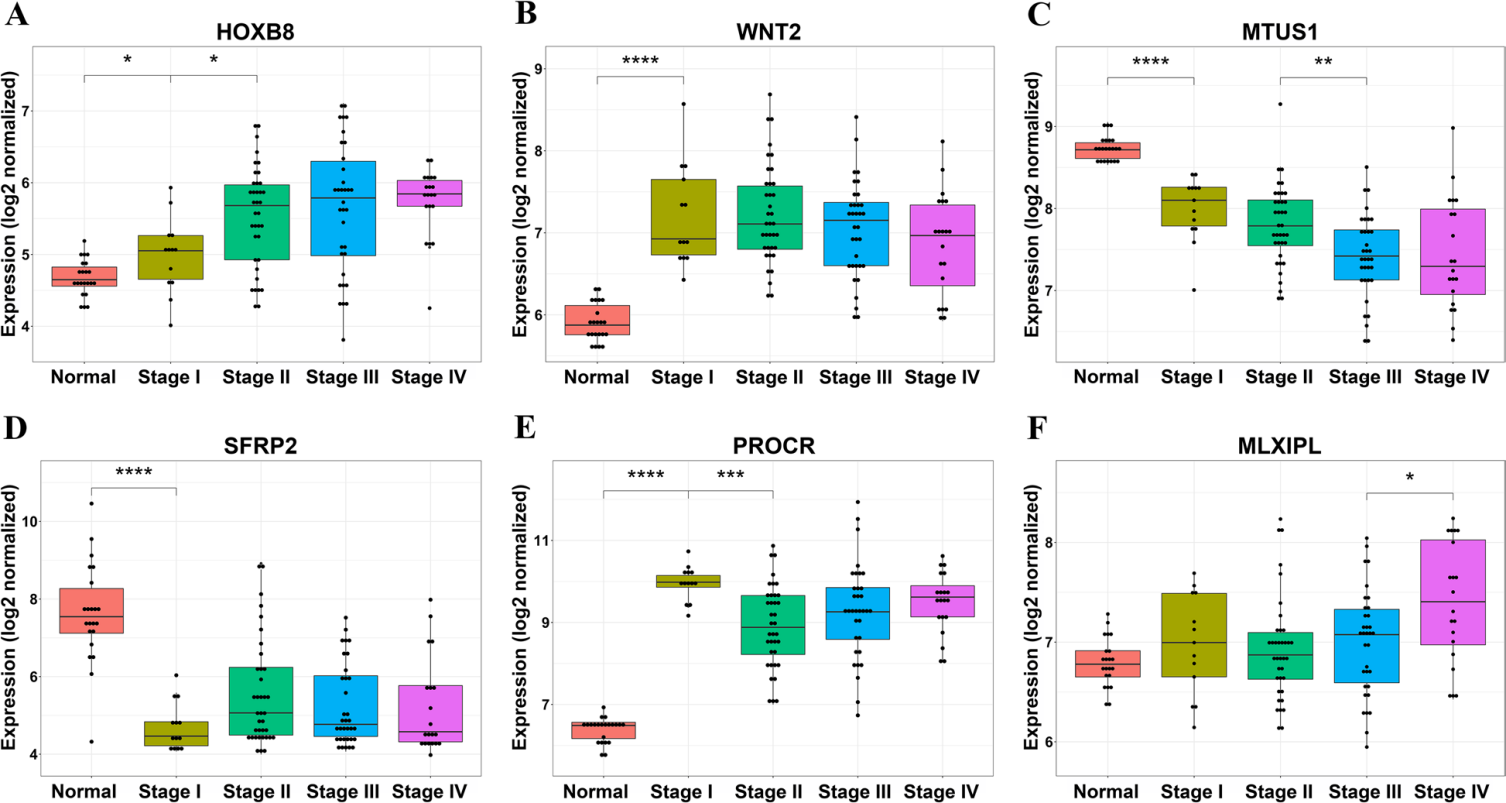
Biomarkers. **A**-**D** Boxplots for 4 biomarkers from STEM analysis and **E–F** boxplots for 2 stage-specific biomarkers, consistent with GEPIA2 COAD-READ cohort results. Each color indicates one stage and dots show the expressions of biomarker gene for patients in every stage. **A** HOXB8 with trend (0,1,2,3,4), **B** WNT2 with trend (0,1,1,1,1), **C** MTUS1 with trend (0,-1,-2,-3,-4), **D** SFRP2 with trend (0,-1,-1,-1,-1), **E** PROCR, stage I-specific biomarker, and **F** MLXIPL, stage IV-specific biomarker

We selected 60 model profiles and the maximum unit change of 1 for the STEM analysis (see Materials and Methods). Most of the genes were clustered in two main trends, (0,1,1,1,1) and (0,-1,-1,-1,-1), implying that the expression of genes changed extensively up or down from the normal condition but with little or no difference across stages I-IV (Figs. 4A-D). The trends identified were consistent with TCGA COAD-READ cohort results from GEPIA2 (Additional file 2: Figures S2A-D). Additional file 1: Tables S12 through S17 list the genes belonging to each trend.

We highlighted some genes which exhibit the aforementioned trends including *HOXB8,* with monotonically increasing expression from normal through the cancer stages (Fig. 4A). Studies have shown that knockdown of *HOXB8* inhibits cellular proliferation and invasion in vitro, as well as carcinogenesis and metastasis in vivo. *HOXB8* has been suggested as an independent prognostic factor in CRC [44, 45]. Likewise, *WNT2*, an oncogene, exhibited an increasing STEM trend and was over-expressed in CRC (Fig. 4B), across stages, compared to normal tissues. *WNT2* is known to be involved in canonical Wnt signaling activation during CRC tumorigenesis, and has been suggested to enhance tumor growth and the invasion in a paracrine fashion [46, 47]. *WNT2* has been previously identified as a stool marker with a sensitivity of 74–78% and specificity of 88–89% [48, 49]. *MTUS1* expression (Fig. 4C), was significantly down-regulated in human colon cancer tissues and has been documented in earlier studies [50]. It has been suggested to be involved in the loss of proliferative control in human colon cancer via its interference of ERK2 pathway activation [51]. *SFRP2* gene, located upstream of the canonical Wnt signaling pathway, was also found to be suppressed across all stages [52]. *SFRP2* was the first reported DNA methylation marker in stool with a sensitivity of 32.1–94.2% and specificity of 54–100% [53]. DNA hypermethylation of *SFRP2* leads to the downregulation of the gene expression, inhibition of gene function and promotion of CRC [48]. GEPIA2 was additionally used to generate disease-free survival (DFS) plots of the four biomarkers identified by STEM analysis (Additional file 2: Figures S3A-D). DFS plot of *HOXB8* confirmed that high expression of this genes was associated with poor disease-free survival of patients with CRC.

#### Stage-specific biomarkers

Biomarkers specific to each stage were identified as the intersection of four sets of DEGs between that stage and other stages with *p*-value ≤ 0.05. In total, 110 potential stage-specific biomarkers including 41 for stage I, 21 for stage II, 8 for stage III, and 40 for stage IV were identified (listed in Additional file 1: Table S18). *p*-values for 10 comparisons (e.g. normal vs stage I) for all 110 potential biomarkers were listed in Additional file 1: Table S19. Figures 4E and 4F show boxplots for *PROCR*, a stage I-specific biomarker and *MLXIPL* (*ChREBP*), a stage IV-specific biomarker, respectively. The trends for these two biomarkers identified were consistent with TCGA COAD-READ cohort results obtained through GEPIA2 (Additional file 2: Figures S2E-F). High expression of *PROCR* and *MLXIPL* was associated with poor disease-free survival of CRC patients (Additional file 2: Figures S3E-F). Through immunohistochemistry, it has been shown that *PROCR* overexpressed in CRC epithelial tumor cells [54]. This upregulation is caused by gene amplification and DNA hypomethylation and occurs in concert with a cohort of neighboring genes on chromosome locus 20q [55]. Studies have shown that *ChREBP* mRNA and protein expression levels are significantly increased in colon cancer tissues compared to normal tissues [56]. Their expression positively correlated with colon malignancy and was suggested to contribute to cell proliferation. Given its functional roles in CRC, and its distinct expression with stage IV, we propose that *ChREBP* could serve as a clinically useful biomarker.

The results presented above were based on an unsupervised analysis at a global network level. We additionally carried out a more focused analysis, emphasizing key drivers of CRC.

### Evolution of subnetwork of key genes and their first neighbors across different stages

We performed a supervised analysis with 10 key genes with known roles in CRC (see Materials and Methods). The key genes were *TP53*, *APC*, *KRAS*, *BRAF*, *PIK3CA*, *EGFR*, *MLH1*, *TGFBR2*, *PTEN*, and *SMAD4*. The union of key genes and their first neighbors from STRING-db yielded 188 unique genes of which 162 were present within our master list of genes.

**Fig. 5:**
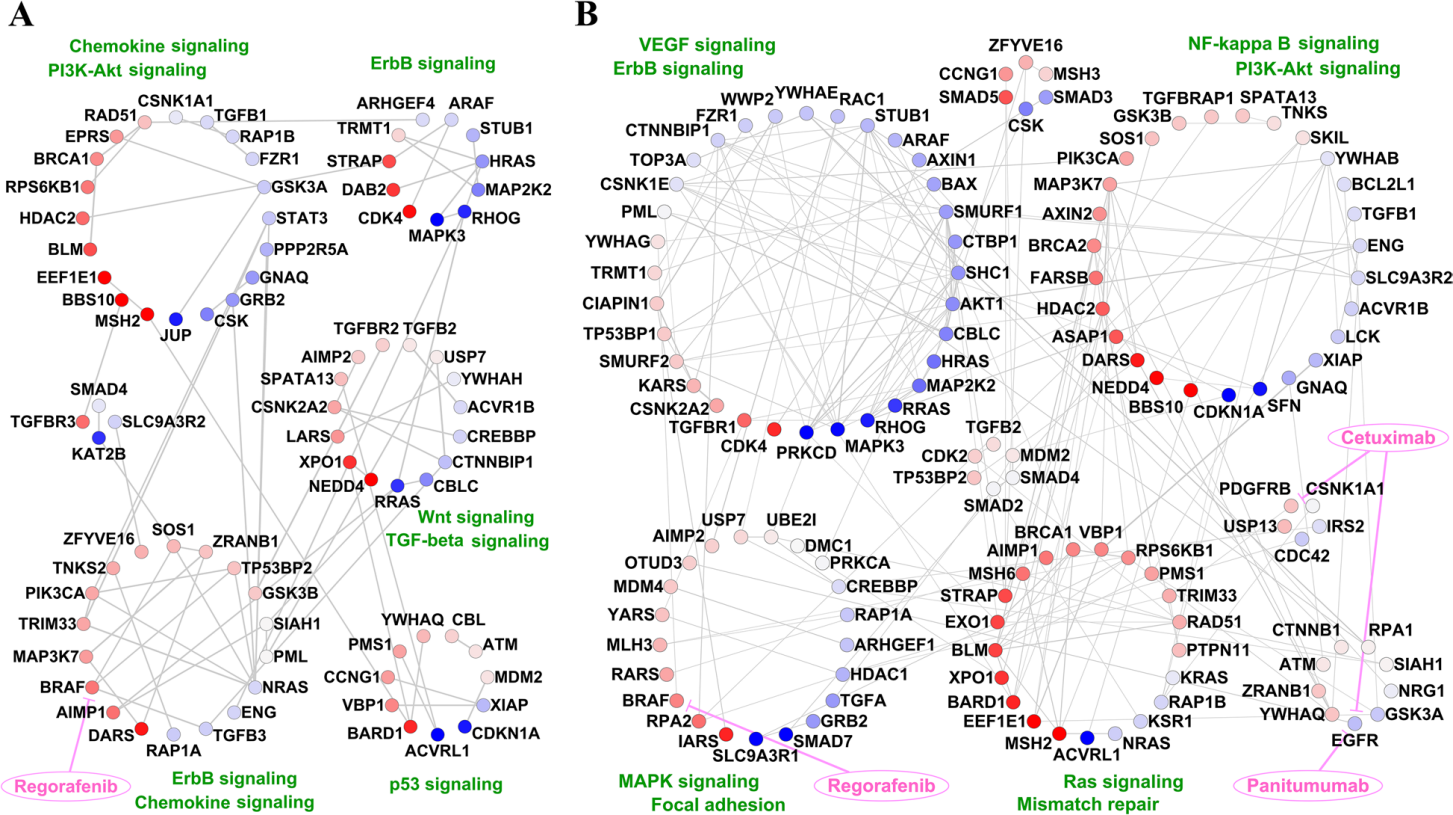
Subnetwork of 162 genes in stages I and II. **A** Stage I-specific network and **B** Stage II-specific network. Nodes of each subnetwork are grouped together based on the communities they belonged to in the stage-specific networks and colored based on the value of log2FC between that stage and normal: dark blue (log2FC of -2), to white (0) to dark red (2). The width of edges shows the strength of connections based on PCC between them. The thicker the edges are, the larger the PCC between the nodes is

The subnetworks of 162 unique genes in stages I- and II-specific networks are shown in Figs. 5A and 5B, respectively. The subnetworks from stages III- and IV-specific networks are shown in Additional file 2: Figures S4A and S4B, respectively. The nodes were clustered based on the communities they belonged to in the stage-specific networks described in the earlier sections. The subnetwork of stage I was more sparse but with stronger edge weights since the stage I-specific network had fewer and stronger edge weights (PCC ≥ 0.8009) than other stages. We observed these networks to be enriched for several drug targets including *BRAF*, *EGFR*, and *PDGFRB*, and several signaling pathways including Chemokine, PI3K-Akt, ErbB, Ras, TGF-beta, Wnt, p53, NF-kappa B, VEGF and MAPK (Fig. 5). The subcommunities of both subnetworks included both up- and down-regulated genes. For instance, Fig. 5A highlights a subcommunity in stage I enriched for several up-regulated genes associated with Chemokine and ErbB signaling pathways, both with known roles in cancer etiology [57, 58]. Likewise, there was a subcommunity in stage II, shown in Fig. 5B, with genes mostly up-regulated and enriched for Ras signaling and mismatch repair pathways. We also detected a subcommunity within stage II with genes mostly down-regulated (Fig. 5B) and enriched for pathways such as ErbB and VEGF signaling. VEGF family members play an essential role in tumor-associated angiogenesis, tissue infiltration, and metastasis formation [59].

These subnetworks all showed differences in connectivity patterns for key genes. For example, *EGFR*, whose degree was zero in all subnetworks except for stage II, is known to play a critical role in oncogenesis, particularly in colon cancer development and is a potential target for therapy [60]. We identified that its expression was down-regulated in the subnetwork of stage II and was connected to *OTUD3* (a tumor promoter in lung cancer). *EGFR* also serves as a drug target for Cetuximab and Panitumumab. *BRAF*, another key player in CRC was up-regulated across all cancer stages compared to normal, yet had distinct connectivity patterns across different stages. *BRAF* was connected to *TGFB3*, *TP53BP2*, and *SOS1* in the subnetwork of stage I. Although the stage II-specific network had more edges compared to stage I-specific network, *BRAF* was connected to only one gene, *YWHAG*, in the subnetwork of stage II. The chemotherapy drug for CRC, Regorafenib, targets *BRAF* and modulates the activity of its protein.

Finally, we sought to understand the functional mechanisms for some of the current drugs used in CRC treatment in the context of our current analysis and identify if any temporal variation in gene-expression of the drug-targets may indicate stage-specificity of the drugs.

### Drug-Target-PPI network

**Fig. 6:**
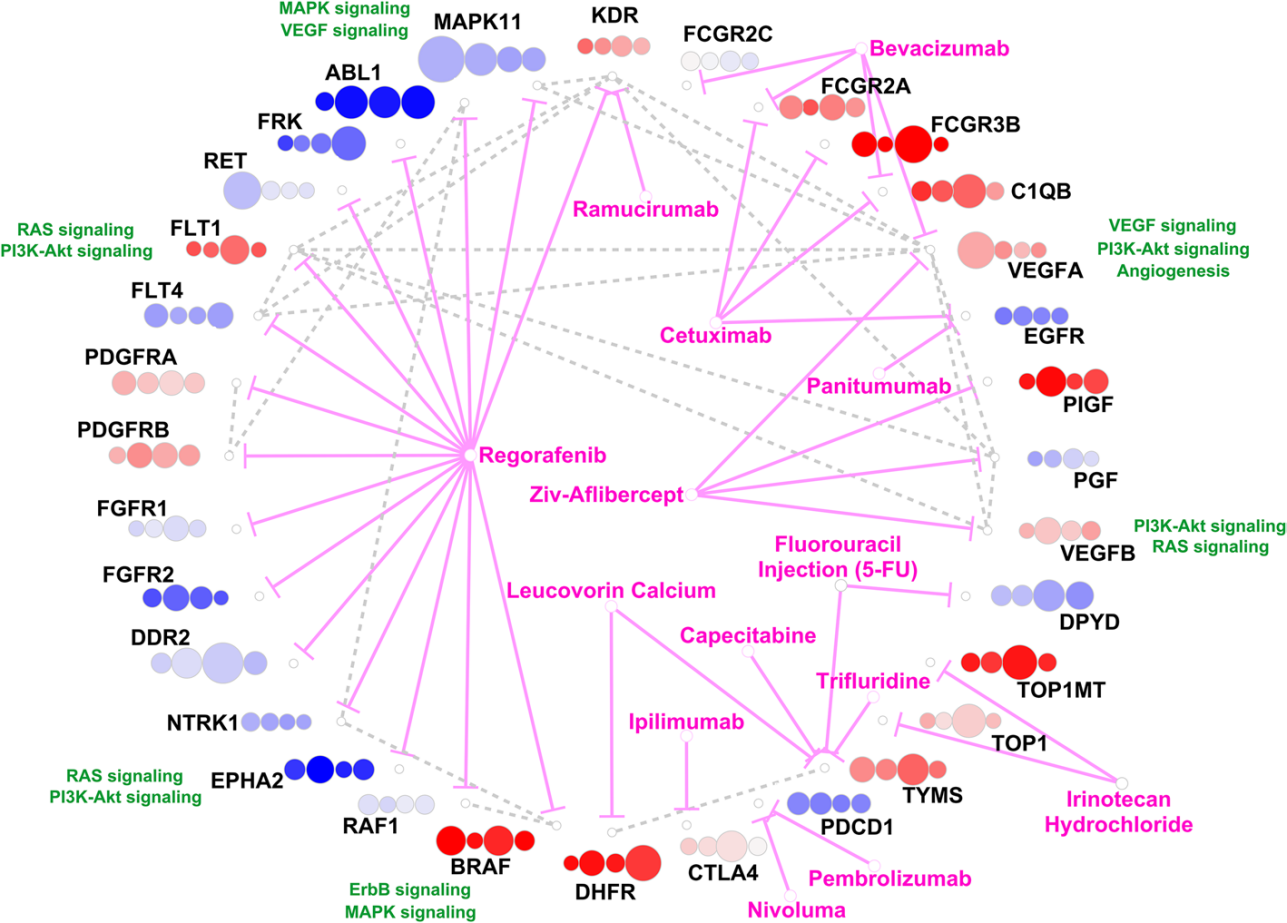
Drug-Target-PPI network for CRC. Fourteen Drugs approved by FDA for treating CRC (mainly when the cancer metastasize) are used to construct this network; the drug nodes are shown in the center area. The target genes have been found from DrugBank. For each target gene node, four circles are associated with that gene corresponding to four stages I-IV, and are colored based on the log2FC values between a stage and normal (dark blue (log2FC of -1) to white (0) to dark red (log2 FC of 1)). The size of each circle represents the sum of the weights of edges connected to that gene (i.e., gene weights) in each stage. For example, *MAPK11* weight is greater in stage I as compared to that in other stages. PPI edges from STRING-db (score threshold ≥ 0.9) are also incorporated in this network by dashed grey-lines between the genes. Some important pathways are also shown. For select functionally important genes, the related functions are listed

We identified 14 FDA-approved drugs for CRC from the NCI website and 32 target genes (included in the master list of DEGs) for these 14 drugs from the DrugBank website [61]. There were 20 edges between the target genes based on STRING-db [28]. Figure 6 shows a Drug-Target-PPI network constructed with the approved drugs. Gene weight, the sum of the weights of edges connected to each gene, in each stage-specific network, are shown beside target genes. Some important pathways involving target genes, such as PI3K-Akt or Ras signaling, are also highlighted in the figure. We can see that the weight of different genes changes across the four stages extensively. log2FC (with respect to normal) for genes also changes albeit to a lesser degree.

Several targets of Regorafenib, a popular CRC drug, were found to be differentially regulated within our networks (Fig. 6). Studies have shown that Regorafenib targets kinases involved in tumor angiogenesis (e.g. *VEGFR1*/*2*/*3*, *FGFR1*/*2*), proliferation (e.g. *MAPK11*, *RET*), tumor microenvironment and metastasis [62, 63]. It can also disrupt tumor immunity through inhibition of *CSF-1R*, important for macrophage differentiation and survival [64]. Out of its targets, *MAPK11* and *RET* were both down-regulated and had greater weights in early stages. *MAPK11* is a member of protein kinases family involved in several cellular processes, including cell proliferation or differentiation. It was also enriched for MAPK and VEGF signaling pathways. *RET*, as a member of the tyrosine protein kinases family, has been identified as a novel tumor suppressor gene in the colon which can reduce apoptosis and is considered as a target for CRC treatment [65, 66]. There were also some targets for Regorafenib with larger weights in later stages, such as *FLT1* and *DDR2*. *FLT1*, a member of the vascular endothelial growth factor receptor (VEGFR) family, was up-regulated in CRC and strongly connected (PPI edge) to three ligands, namely, *VEGFA*, *VEGFB* and *PGF* [67]. *DDR2*, down-regulated in CRC, is considered a critical regulator of cancer invasion and an attractive therapeutic target in metastatic CRC (mCRC) [68].

Two up-regulated and highly connected genes in this network, *VEGFA* and *VEGFB* are targets of the drug Ziv-Aflibercept, and participated in Ras and PI3K-Akt signaling pathways with known roles in CRC progression. *VEGFA* had larger weights in stage I whereas *VEGFB* had larger weights in stage II. *TYMS* (part of the Folate‐mediated one‐carbon metabolism pathway) is a crucial player of DNA methylation and repair and a critical target for Fluorouracil Injection (5-FU) drug, used in CRC treatment [69]. Studies have shown that *TYMS* is highly expressed in patients with CRC and might be used as a predictor for efficacy of chemotherapy [70]. Its weight was higher in stage III than in other stages. *TOP1* and *TOP1MT*, both up-regulated in CRC, had also greater weight in stage III and were targets for Irinotecan Hydrochloride which is one of the key drugs for the treatment of mCRC [71].

Besides Regorafenib, two other drugs, Cetuximab and Bevacizumab, commonly used in treating CRC also showed several targets enriched within our networks. *C1QB*, a target for both of those drugs, was up-regulated with greater weights in stage III. Cetuximab blocks ligand-induced receptor signaling and modulates tumor-cell growth by binding to the extracellular domain of *EGFR*. Studies have also shown that Cetuximab improves overall survival and progression-free survival and preserves quality-of-life measures in CRC patients in whom other treatments have failed [72]. Bevacizumab, which binds to and targets *VEGF*, also has demonstrated improved overall survival for patients with mCRC [73].

The pathogenesis of CRC is yet to be fully understood. In this study we detected a few potential biomarkers which were further validated *in-silico*, using a large cohort database (TCGA COAD-READ). However, further experimental validation is required to decipher their pathology-associated mechanisms. Additionally, we were limited by the unequal number of patient samples across stages and lacked sufficient clinical metadata to support downstream survival analysis. Nevertheless, the modular-network-based approach presented in this work will be useful for understanding mechanisms for disease progression and may contribute to identifying potential targets for disease intervention. In addition, while digital sequencing data are more robust, this microarray analog gene expression data set has been used extensively and our quest was to explore topological network analyses to demonstrate the ability to obtain stage-specific biomarkers and mechanisms. We demonstrate the validity of our conclusions through extant results and additional analyses.

## Conclusion

In this study, we utilized a published transcriptomic data from 128 patients at various stages of CRC to find modular mechanisms potentially causal for progression of CRC from normal to stages I-IV and to find stage-specific biomarkers. We constructed stage-specific networks and identified their communities using the *Louvain* algorithm. Comparing communities of different networks at the topological and functional levels revealed that neighboring stages were more similar to each other than non-neighboring stages. We also carried out the functional analysis at the whole network level for the stage-specific and stage-unique networks by analyzing the enrichment of 24 cancer-related pathways across different stages. For the stage-specific networks, most of the pathways related to CRC such as PI3K-Akt and MAPK signaling pathways were enriched at all stages. However, stage-unique networks revealed functional differences across the stages. For example, MAPK signaling pathway was enriched across stages I-III and Notch signaling pathway (important for metastasis and tumor angiogenesis) was enriched in stages III and IV. We then identified key biomarkers to differentiate between CRC (any stage) and normal using STEM analysis. *WNT2* and *SFRP2* were two biomarkers validated by others in stool DNA and were over-expressed and under-expressed in CRC tissues, respectively. To incorporate legacy knowledge in our analysis, we performed a supervised analysis with 10 key genes related to CRC and their first neighbors based on STRING-db, across different stages. The subnetworks were analyzed to study the progression of cancer across stages. In particular, we identified that *BRAF*, a Ser/Thr kinase that activates MAP kinases, appeared in all subnetworks and was upregulated in stages I-IV as compared to normal. Its connectivity pattern changed across the subnetworks for normal and different stages of CRC. Finally, we constructed a Drug-Target-PPI network enabling us, in the light of present data, to understand the functional mechanisms for some of the current drugs for CRC treatment. We saw that the target gene weights changed across the four stages extensively. For example, *TYMS*, associated with folate-mediated one carbon metabolism and a target for some drugs such as Fluorouracil Injection (5-FU) and Capecitabine, was found to be upregulated in cancer stages with larger weights in stage III than in other stages.

## Supplementary Information

**Additional file 1: Supplementary tables.**  An .xlsx file containing all supplementary tables listed in manuscript as individual sheets.

**Additional file 2: Supplementary figures. **A .pdf file containing all supplementary figures referenced in manuscript.

**Additional file 3. **Supplementary Methods [74]. 

## Data Availability

The dataset analyzed in the current study is available in the Gene Expression Omnibus (GEO) repository, [https://www.ncbi.nlm.nih.gov/geo/query/acc.cgi?acc=GSE21510].

## Abbreviations

CRC: Colorectal Cancer
DAVID: Database for Annotation, Visualization and Integrated Discovery
DEG: Differentially Expressed Gene
DFS: Disease-Free Survival
FDA: Food and Drug Administration
FDR: False Discovery Rate
GEO: Gene Expression Omnibus
GEPIA: Gene Expression Profiling Interactive Analysis
JI: Jaccard Index
KEGG: Kyoto Encyclopedia of Genes and Genomes
mCRC: Metastatic CRC
MI: Mutual Information
NCI: National Cancer Institute
NMI: Normalized Mutual Information
PCA: Principal Component Analysis
PCC: Pearson Correlation Coefficient
PPI: Protein–Protein Interaction
RMA: Robust Multi-array Average
STEM: Short Time-series Expression Miner
TCGA: The Cancer Genome Atlas
t-SNE: t-Distributed Stochastic Embedding
VEGFR: Vascular Endothelial Growth Factor Receptor
