# Supplementary material for: Modular and mechanistic changes across stages of colorectal cancer
Source: BMC Cancer. 2022 Apr 21;22:436. doi: 10.1186/s12885-022-09479-3 (PMC9022252; doi:10.1186/s12885-022-09479-3)
Supplement: Supplementary file 2 — Additional file 2: Supplementary figures. A .pdf file containing all supplementary figures referenced in manuscript. [file 12885_2022_9479_MOESM2_ESM.pdf]

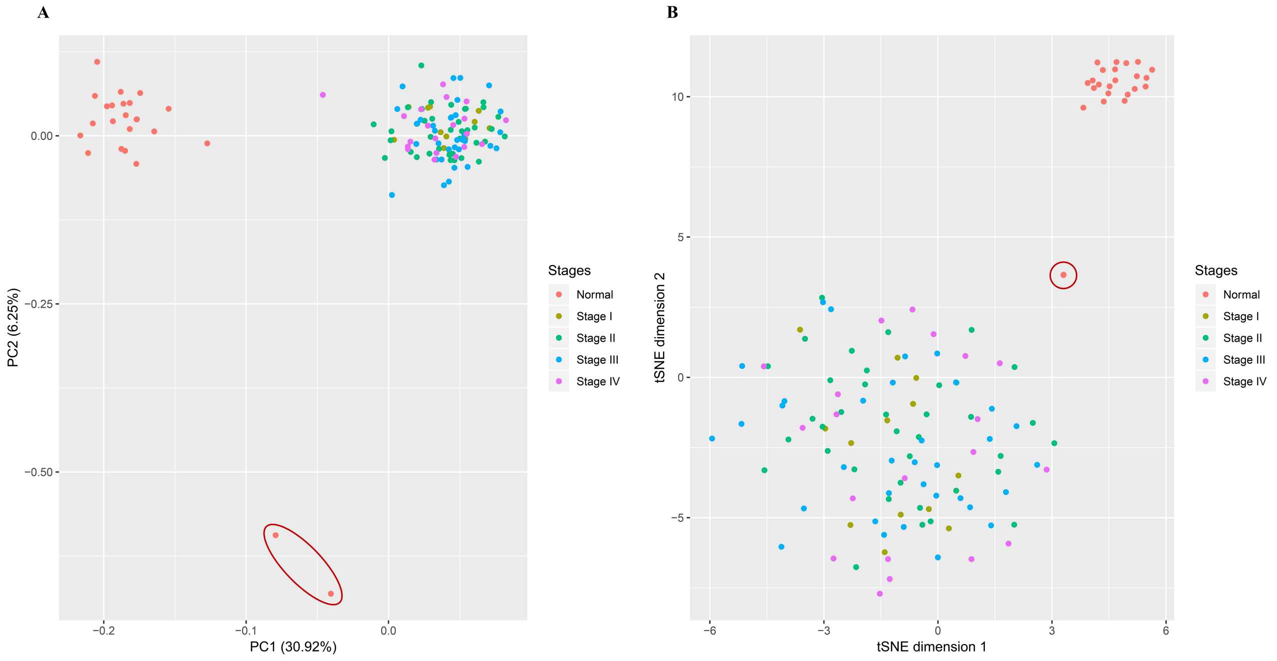

Figure S1. Dimensionality reduction techniques applied on 41834 probe IDs and 128 samples including 13 patients in stage I, 37 patients in stage II, 34 patients in stage III, 20 patients in stage IV, and 24 normal samples. **A.** PC1 vs PC2. There are two outliers in normal, in a red circle. **B.** First two dimensions of t-SNE method. There are two outliers in normal which are very close to each other, highlighted in a red circle. These two outliers are similar to the outliers found by PCA analysis.

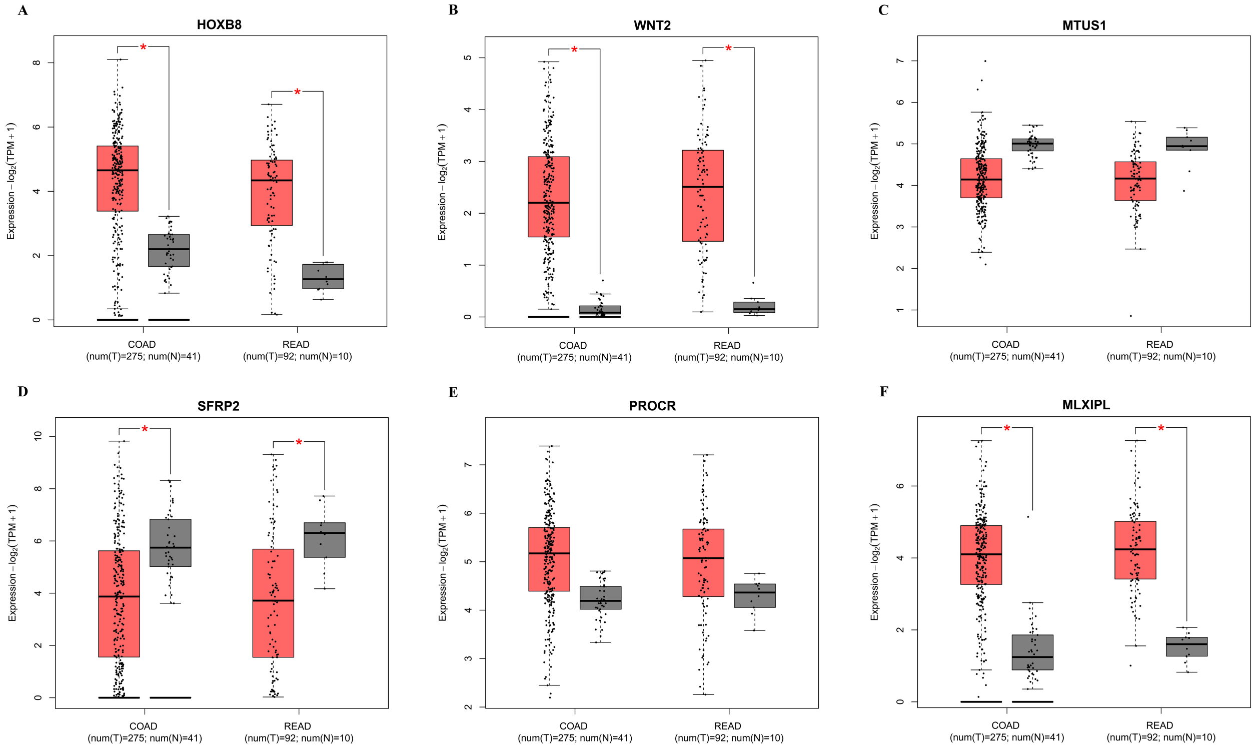

Figure S2. Gene expression boxplots of 6 biomarkers using TCGA COAD-READ cohort through GEPIA2. **A-D.** Boxplots for 4 biomarkers from STEM analysis. **E-F.** Boxplots for 2 stage-specific biomarkers. Red rectangles represent tumor and gray rectangles represent normal in each boxplot.

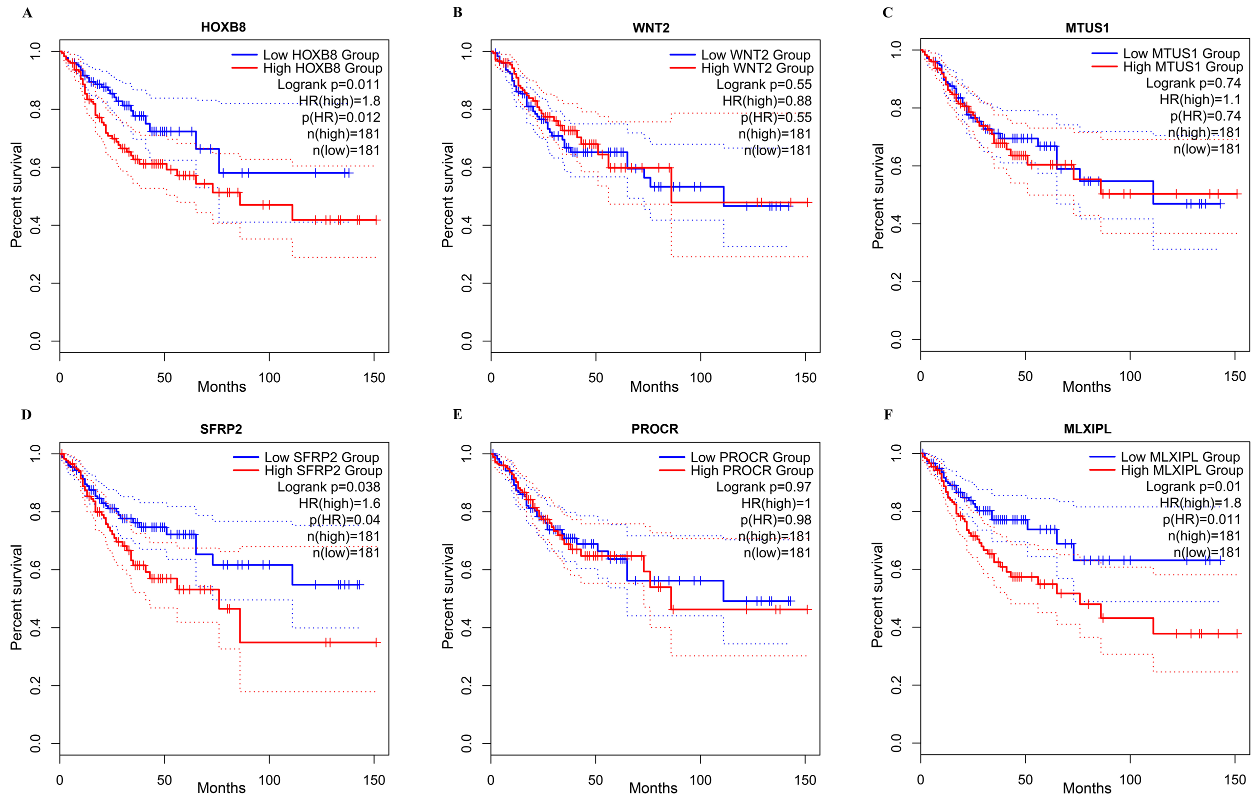

Figure S3. Kaplan-Meier curves for Disease-Free Survival (DFS) using TCGA COAD-READ cohort through GEPIA2. **A-D.** DFS plots for 4 biomarkers from STEM analysis. **E-F.** DFS plots for 2 stage-specific biomarkers. Red lines represent the samples with highly expressed genes and blue lines represent the samples with lowly expressed genes. Dotted line shows a 95% confidence interval. HR: hazard ratio.

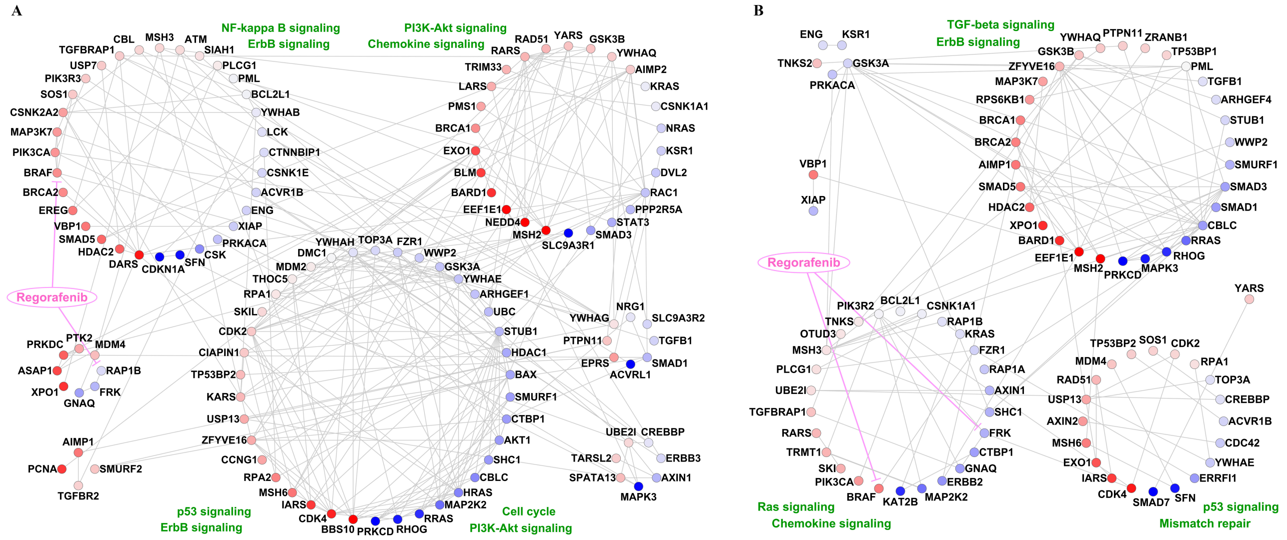

Figure S4. Subnetwork of 162 genes in **A.** stage III-specific network and **B.** stage IV-specific network. Nodes of each subnetwork are grouped together based on the communities they belonged to in the stage-specific networks and colored based on the value of log2FC between that stage and normal: dark blue (log2FC of -2), to white (0) to dark red (2). The width of edges shows the strength of connections based on PCC between them. The thicker the edges are, the larger the PCC between the nodes is.
