# Supplementary material for: Modular and mechanistic changes across stages of colorectal cancer
Source: BMC Cancer. 2022 Apr 21;22:436. doi: 10.1186/s12885-022-09479-3 (PMC9022252; doi:10.1186/s12885-022-09479-3)
Supplement: Supplementary file 3 — Additional file 3. Supplementary Methods [74]. [file 12885_2022_9479_MOESM3_ESM.docx]

# Supplementary Methods

## t-Distributed Stochastic Neighbor Embedding (t-SNE)

t-SNE is a nonlinear dimensionality reduction algorithm. It maps multi-dimensional data to a few (two or more) dimensions, which can be easily visualized [[20](#_ENREF_20)].

The algorithm comprises three steps as follows:

1. In the 1^st^ step, t-SNE measures similarities between points in the high dimensional space. For each data point, $x_{i}$, the algorithm centers a Gaussian distribution over that point and measures the density of all points under the distribution and renormalizes the densities. This gives a set of probabilities, $p_{ij}$, which is proportional to the similarity and is calculated by Equation S1 [[20](#_ENREF_20)].

| $p_{ij}=\frac{exp(-\left\Vert x_{i}-x_{j} \right\Vert^{2}/2\sigma^{2})}{\sum_{k\neq l} exp(-\left\Vert x_{k}-x_{l} \right\Vert^{2}/2\sigma^{2})}$ |  | (S1) |
| --- | --- | --- |

where $x$’s are the points in the high-dimensional space and $\sigma$ is the variance of the Gaussian distribution.

1. This step is similar to step 1 but here, the algorithm uses a student-t distribution rather than a Gaussian distribution to compute the similarities, $q_{ij}$, in the low-dimensional space (two or more). Mathematically, $q_{ij}$ is given by.

| $q_{ij}=\frac{exp(-\left\Vert y_{i}-y_{j} \right\Vert^{2}/2\sigma^{2})}{\sum_{k\neq l} exp(-\left\Vert y_{k}-y_{l} \right\Vert^{2}/2\sigma^{2})}$ |  | (S2) |
| --- | --- | --- |

where $y$’s are the points in the low-dimensional space.

1. In the last step, the algorithm matches the two probability distributions in the high and low dimensional spaces, by minimizing the Kullback-Leibler (KL) divergence. The KL divergence (cost function) is given by Equation S3 and the algorithm uses gradient descent to minimize it [[20](#_ENREF_20)].

| $C= KL(P\vert\vert Q)=\sum_{i} \sum_{j} p_{ij}\log\frac{p_{ij}}{q_{ij}}$ |  | (S3) |
| --- | --- | --- |

## Normalized Mutual Information (NMI) metric

NMI is a metric to calculate the similarity between two groups. It is the normalized form of Mutual Information (MI). MI measures similarity between two methods (stages) and is given by [[74](#_ENREF_74)]:

| $MI\left( A,B \right)=\sum_{i=1}^{c_{A}} \sum_{j=1}^{c_{B}} \frac{N_{ij}}{N}\log\left( \frac{N_{ij}N}{N_{i.}N_{.j}} \right)$ | (S4) |
| --- | --- |

where, *A* and *B* are the stages being compared. Denote by *N_i_.* the number of nodes (genes) in community *i* of stage *A* and *N_.j_* the number of nodes in community *j* in stage *B*. *N_ij_*, is the number of nodes in both community *i* of stage *A* and community *j* of stage *B*.

Then, NMI between stages *A* and *B* is calculated as [[22](#_ENREF_22)]:

| $NMI(A,B)=\frac{-2\sum_{i=1}^{c_{A}} \sum_{j=1}^{c_{B}} \frac{N_{ij}}{N}\log\left( \frac{N_{ij}N}{N_{i.}N_{.j}} \right)}{\sum_{i=1}^{c_{A}} N_{i.}\log\left( \frac{N_{i.}}{N} \right)+\sum_{j=1}^{c_{B}} N_{.j}\log\left( \frac{N_{.j}}{N} \right)}$ | (S5) |
| --- | --- |

## Community detection algorithm, *Louvain*

As described in the main manuscript, *Louvain* algorithm detects communities in networks by maximizing modularity, calculated by Equation S6:

| $Q=\frac{1}{2m}\sum_{i,j} \left[ A_{ij}-\frac{k_{i}k_{j}}{2m} \right]\delta\left( c_{i}, c_{j} \right)$ |  | (S6) |
| --- | --- | --- |

where *A_ij_* is the weight of the edge between node *i* and *j* (is equal to 1 when all edges have the same weight), *k_i_* is the sum of the weights of the edges attached to node *i* or degree of node *i*, *c_i_* is the community to which node *i* belongs to and the *δ* function is defined as *δ*(*u,v*) *=* 1 if *u = v* and 0 otherwise. *m* is the total number of edges in an unweighted network and the sum of the weights of all edges in a weighted network.

The algorithm is divided in two phases, which are repeated iteratively. First phase is to assign a different community to each node of the network. So, in the beginning, there are as many communities as there are nodes. Then, the gain of modularity (Equation S6) is calculated for removing node *i* from its community and placing it in one of its neighboring communities. The gain of modularity in moving node *i* into a community *C* can be computed by:

| $\Delta Q=\left[ \frac{\sum_{in}+k_{i,in}}{2m}-\left( \frac{\sum_{tot}+k_{i}}{2m} \right)^{2} \right]-\left[ \frac{\sum_{in}}{2m}-\left( \frac{\sum_{tot}}{2m} \right)^{2}-\left( \frac{k_{i}}{2m} \right)^{2} \right]$ |  | (S7) |
| --- | --- | --- |

where Ʃ*_in_* is the sum of the weights (or count for un-weighted networks) of the edges inside *C*, Ʃ*_tot_* is the sum of the weights of the edges incident to nodes in *C*, *k_i_* is the sum of the weights of the edges incident to nodes *i* (degree of *i*), *k_i,in_* is the sum of the weights of the edges from *i* to nodes in *C*. If the gain is positive, the node *i* is placed in the community for which the gain is maximum. This process is applied repeatedly for all nodes until no further improvement can be achieved.

The second phase is to build a network whose nodes are now the communities detected in the first phase. In order to perform that, the weights of the edges between the new nodes are given by the sum of the weights of the edges between nodes in the corresponding two communities. Edges between nodes of the same community result in self-loops for this community in the new network. When this phase is completed, the first phase of the algorithm is reapplied to the new network. The combination of these two phases is referred to as a “pass”. The passes are iterated until a maximum of modularity is reached [[14](#_ENREF_14)].

## STEM algorithm

Short Time-series Expression Miner (STEM) is an algorithm designed for clustering short time expression data. First, the algorithm selects a set of potential profiles and then genes are assigned to the profile that best represents their trend among the pre-selected profiles.

To define a set of model profiles, the user must specify a parameter *c* that controls the amount of maximum change a gene has between successive time points. For example, if *c* is 2, then a gene can go up either one or two units, stay the same or go down either one or two units between successive time points. So, for *n* time points, this strategy would give ${(2c+1)}^{n-1}$ distinct profiles. Since most of these profiles are likely to be sparsely populated, a subset of them, *R,* has to be selected, such that the minimum distance between any two profiles in *R*, namely *p_1_* and *p_2_* is maximized. This can be formulized as:

| $\text{max}_{R\subset P,\left\vert R \right\vert=m}\text{min}_{p_{1},p_{2}\in R}d(p_{1},p_{2})$ |  | (S8) |
| --- | --- | --- |

where *P* represents the total set of possible profiles, *d* is a distance metric, and *m* is the size of *R* (i.e., |*R*| = *m*). A greedy algorithm is used to calculate *R*. The algorithm starts with one of the two extreme profiles and in each iteration, selects the profile that is farthest from all profiles located in *R* so far. This process is repeated until *m* profiles have been selected.

In the next step, each gene *g* ∈*G* is assigned to a model profile *m_i_* in the set of profiles *M*, such that *d*(*e_g_*, *m_i_*) is the minimum over all *m*’s, where *e_g_* is the temporal expression profile for gene *g*. If the above equation is minimized by more than one profile (*h* > 1), then *g* is assigned to all those profiles, but the assignments is weighted as 1*/h*. Further details about the algorithm can be found elsewhere [[15](#_ENREF_15), [16](#_ENREF_16)].
